# Arc protein expression after unilateral intracranial self-stimulation of the medial forebrain bundle is upregulated in specific nuclei of memory-related areas

**DOI:** 10.1186/s12868-018-0449-5

**Published:** 2018-08-08

**Authors:** Elisabet Kádár, Eva Vico Varela, Laura Aldavert-Vera, Gemma Huguet, Ignacio Morgado-Bernal, Pilar Segura-Torres

**Affiliations:** 1 Departament de Psicobiologia i de Metodologia de les Ciències de la Salut, Institut de Neurociències, Universitat Autónoma de Barcelona, 08193 Bellaterra, Barcelona, Spain; 2 Departament de Biologia, Universitat de Girona, 17071 Girona, Spain; 3 Douglas Mental Health University Institute, McGill University, Montreal, QC H4H 1R3 Canada; 4 Department of Biology, Sciences Faculty, University of Girona, C/Mª Aurèlia Capmany 40, Camous Montilivi, 17003 Girona, Spain

**Keywords:** Arc, Medial forebrain bundle, Intracranial self-stimulation, Memory, Hippocampus, Amygdala, Thalamus, Habenula

## Abstract

**Background:**

Intracranial Self-Stimulation (ICSS) of the medial forebrain bundle (MFB) is a deep brain stimulation procedure, which has a powerful enhancement effect on explicit and implicit memory. However, the downstream synaptic plasticity events of MFB-ICSS in memory related areas have not been described thoroughly. This study complements previous work studying the effect of MFB-ICSS on the expression of the activity-regulated cytoskeleton-associated (Arc) protein, which has been widely established as a synaptic plasticity marker. We provide new integrated measurements from memory related regions and take possible regional hemispheric differences into consideration.

**Results:**

Arc protein expression levels were analyzed 4.5 h after MFB-ICSS by immunohistochemistry in the hippocampus, habenula, and memory related amygdalar and thalamic nuclei, in both the ipsilateral and contralateral hemispheres to the stimulating electrode location. MFB-ICSS was performed using the same paradigm which has previously been shown to facilitate memory. Our findings illustrate that MFB-ICSS upregulates the expression of Arc protein in the oriens and radiatum layers of ipsilateral CA1 and contralateral CA3 hippocampal regions; the hilus bilaterally, the lateral amygdala and dorsolateral thalamic areas as well as the central medial thalamic nucleus. In contrast, the central amygdala, mediodorsal and paraventricular thalamic nuclei, and the habenular complex did not show changes in Arc expression after MFB-ICSS.

**Conclusions:**

Our results expand our knowledge of which specific memory related areas MFB-ICSS activates and, motivates the definition of three functionally separate groups according to their Arc-related synaptic plasticity response: (1) the hippocampus and dorsolateral thalamic area, (2) the central medial thalamic area and (3) the lateral amygdala.

**Electronic supplementary material:**

The online version of this article (10.1186/s12868-018-0449-5) contains supplementary material, which is available to authorized users.

## Background

Deep brain stimulation (DBS), an electrical current delivered through stereotactically implanted electrodes into specific areas of the brain, is a promising therapeutic option for patients with neurological and psychiatric diseases. To date, DBS has been successfully applied to alleviate movement disorders, such as Parkinson’s disease [1]. DBS is now being considered for use as a treatment for neurodegenerative disorders associated with memory impairments such as Alzheimer’s disease [2]. However, the mechanisms of action of how DBS affects memory are not yet fully known.

Studies with laboratory animals are required to explore potential targets and to analyze the underlying cellular and molecular brain changes of DBS. Recent studies indicate that bilateral DBS of the fornix/hypothalamic area drives neural activity in the cortico-hippocampal memory circuit with a significant reversal of the impaired cortical glucose utilization observed in patients with Alzheimer’s disease [3, 4]. Further experiments in rats have established that forniceal DBS (F-DBS) induces increased hippocampal expression of c-Fos protein, some neurotrophic factors such as BDNF, and other synaptic plasticity markers [5], which are prominent molecular correlates of memory consolidation. These results are consistent with findings from our group which reveal a memory improvement after stimulating the medial forebrain bundle (MFB), the most important pathway of the brain reward system, which passes through the lateral hypothalamus (LH). Activation of brain areas belonging to the reward system generates positive reinforcement, and actions that are positively reinforced are more likely to be repeated than those that do not. Intracranial self-stimulation (ICSS) is a DBS procedure in which subjects self-administer electrical stimulation to brain reward areas by performing an instrumental response, such as pressing a lever in a Skinner box. The use of ICSS in animals allows us to unequivocally ensure that stimulation activates the reward system in a functional manner. In a similar way to F-DBS, ICSS of the LH has a reliable enhancing effect on hippocampus-dependent explicit memory [6–8]. However, while F-DBS affects fornix fibers, ICSS of the LH activates the MFB. Due to the extensive network of the MFB connects, this causes a widespread state of arousal and simultaneous activation of many areas, some of which are associated with different memory systems [9]. This could explain the wide therapeutic effects that MFB-ICSS appears to have in relation to different memory types. In addition to explicit memory, MFB-ICSS also improves performance in implicit memory tasks [10–12]. Most notable are the effects on emotional memory as measured by amygdala-dependent active avoidance tasks, in which ICSS reverses the memory deficit caused by aging [13, 14] and/or brain damage [15, 16].

Consistent with the broad effect of MFB-ICSS on different types of learning tasks, c-Fos expression results suggest ICSS activates multiple regions related to different memory systems, such as the amygdala, the dorsal striatum, the hippocampus and the prefrontal cortex [10, 17–19]. Additionally, MFB-ICSS induces differential time course mRNA expression of some synaptic plasticity-related genes including Arc in the amygdala and the hippocampus [18, 19]. ICSS-treated rats showing a facilitated spatial memory have also long-lasting structural changes including extended dendritic arborization of pyramidal CA1 neurons [6]. Thus, based on the fact that Arc protein is a well-known marker of synaptic plasticity events associated with the memory consolidation process [20] and is also involved in long-term spine enlargement [21], we initially set out to determine the effect of MFB-ICSS has on Arc protein levels in the different sub-regions of the hippocampus and in the retrosplenial cortex. We observed that after 4.5 h of unilateral MFB-ICSS, Arc protein levels increased significantly in the CA1 and DG hippocampal subfields ipsilateral to the stimulated hemisphere [22] but at this time contralateral regions were not analyzed. We also observed that MFB-ICSS increased Arc expression in the granular retrosplenial cortex (RSC), a hippocampus-related region involved in long-lasting memory storage [23]. This study showed a possible lateralization of the ICSS effects on Arc protein levels since changes were only observed in the ipsilateral, but not the contralateral hemisphere.

The current study provides new integrated data of the synaptic plasticity effects of the memory enhancing MFB-ICSS treatment on additional regions related to different memory systems and analyzes hemisphere differences. Arc protein expression was examined by immunohistochemistry in both ipsilateral and contralateral hemispheres 4.5 h after MFB-ICSS in each subject. The studied areas include (1) the hippocampal subfields, involved in spatial memory encoding and consolidation; (2) amygdalar nuclei involved in implicit emotional memory, including the associative lateral amygdala (LA) and the central nucleus (Ce), responsible for the autonomic components of emotions [24]; (3) higher order thalamic nuclei, such as the central medial (CM) and paraventricular (PV) nuclei related to memory through its role in regulation of arousal or attentional levels [25], and the mediodorsal (MD) and dorsolateral (DL) nuclei, considered to be relays to the hippocampus, amygdala and/or associative cortices, as well as being involved in learning and executive functions [26, 27]; and finally (4) the medial and the lateral habenula (MHb and LHb), involved in reward, emotional behavior and cognitive functions [28], which could also be implicated in the circuit in which MFB-ICSS affects memory via the inactivation of structures that project to it [29, 30].

Our findings show that the memory systems engaged by MFB-ICSS include the hilus, the oriens and radiatum layers of CA1 and CA3 hippocampal areas, the LA, DL and CM thalamic nuclei, in accordance with the idea that separate neural systems could operate in parallel to support the effects of ICSS on memory. In addition, a differential hemispheric Arc dependent-synaptic plasticity response to the MFB-ICSS treatment was observed in some of the analyzed regions.

## Methods

### Subjects

Twenty-five male Wistar rats were used in total, with a mean age of 96.20 days (SD = 2.10) at the beginning of the experiment and an average weight of 361 g (SD = 22.7) at the time of surgery. All were bred at our laboratory and fed ad libitum. Animals were individually housed, kept under controlled temperature (20–22 °C), humidity (40–70%) and subjected to a light/dark cycle of 12/12 h.

### Intracranial self-stimulation

#### Stereotactic surgery

A combination of Ketamine (Imalgene, 150 mg/Kg, Merial, Lyon, France) and Xylazine (Rompun 8 mg/kh, Bayer, Barcelona, Spain) was used to induce deep anesthesia in the subjects. Using a digital stereotactic apparatus (Stoelting Co., 51900, IL, USA), all animals were implanted chronically and unilaterally (right hemisphere) with a 150 µm diameter monopolar stainless steel electrode (PlasticsOne, Roanoke, Va, USA). The tip of the electrode was placed at the LH, within the fibers of the medial forebrain bundle, with the incisor bar set at − 2.7 mm below the interaural line and according to coordinates [31]: AP = − 2.56 mm; ML = 1.8 mm and DV = − 8.5 mm, using the cranium surface as a dorsal reference. ICSS electrodes were anchored to the skull with jeweler’s screws and dental cement.

#### ICSS behavior establishment

After a recovery period of 7 days, subjects were randomly distributed in 2 experimental groups, one that received the ICSS treatment (ICSS group) and their respective control without MFB-ICSS (Sham group). In order to establish self-stimulation behavior behavioral shaping was performed on animals from the MFB-ICSS group using a skinner conditioning box (25 × 20 × 20 cm) (Campden Instruments, Ltd.) with a lever situated on one of the lateral walls and a light switching on in a contingent manner to the stimulation train. The stimulation consisted of sinusoidal wave currents, of 0.3 s in duration and 50 Hz in frequency, with an intensity ranging from 50 and 250µA. The procedure included the reinforcement of successive approximations to both the lever and to the required action (lever press) by the animal, while attempting to find the minimum current intensity that gave a stable response of 250 lever presses in 5 min, and self-stimulation until 500 reinforcements were reached. Establishment of the optimal intensity (OI) for each animal was performed in the corresponding ICSS treatment session as described in Segura-Torres et al. 1988 [32].

#### ICSS treatment

Four days after the OI was found, MFB-ICSS treatment was administered. This was done in a single session of 2500 self-administered trains of stimulant current at each particular OI using the same Skinner box where ICSS behavior had been established. The parameters of the stimulation in terms of frequency, intensity and number of trains administered were the same as previous studies since it has been shown to effectively enhance memory, both implicit [33–35] and explicit [6, 8].

Control subjects were placed in the same operant box for 45 min to match the average time it took the ICSS subjects to self-administer the treatment, but without receiving any stimulation (sham session). One of the experimental subjects had to be removed from further analysis at this point in time, due to incomplete treatment.

### Arc Immunolocalization

#### Tissue collection

The animals (ICSS: n = 7, Sham: n = 10) were anesthetized with a lethal dose of pentobarbital (150 mg/kg body weight, i.p.) and perfused transcardially with a solution of 0.1 M phosphate buffer saline (PBS), pH 7.4, followed by a solution of 4% paraformaldehyde in PBS 4,5 h after MFB-ICSS or sham session. This interval has been used previously [22, 23] and chosen as an intermediate point where Arc induction persists after LTP, contextual fear conditioning and reversal learning in a T-maze [20, 21, 36, 37]. Brains were post-fixed in 4% paraformaldehyde in PBS solution for 4 h and then placed in 15% sucrose in PBS for 3 days and 30% sucrose in PBS at 4 °C until they sank. Serial coronal sections of cryopreserved brain (20-μm-thick) were obtained in a cryostat (Cryocut 1800, with 2020 JUNG microtome) at − 20 °C, at coordinates between − 2.50 and − 3.36 AP to Bregma. They were then mounted onto SuperFrost/Plus slides (Menzek-Gläser, Braunschweig, Germany) and stored at − 80 °C until immunohistochemistry staining.

#### Immunohistochemistry

Frozen coronal sections were washed in 0.05% Tween 20 in PBS 0.1 M (Wash Buffer solution), treated in 1% distilled H_2_O_2_ for 15 min and incubated in TNB (0,1 M Tris–HCl pH 7.5, 0,15 M NaCl and 0.5% Blocking Reagent; Perkin Elmer Life Sciences, Inc.) as a blocking solution. Sections were incubated in mouse Anti-Arc antibody (sc-166461, Santa Cruz Biotechnology, Inc.; Santa Cruz, CA, USA; diluted 1:50) for 48 h at 4 °C in a humidified chamber. Later the sections were washed and incubated in Biotinylated anti-mouse IgG antibody (Vector Laboratories Inc.; Burlingame, CA, USA; diluted 1:100) ON at 4 °C. Finally, samples were incubated in Streptavidin-peroxidase (Perkin-Elmer Life Sciences, Inc., diluted 1:100) for 2.5 h at room temperature and washed and incubated in DAB (Fisher) for 10 min. Lastly, sections were dehydrated, mounted and cover slipped. No staining was observed in control slides without the primary or secondary antibodies.

#### Data analysis

Microphotographs were taken with a BX41 Olympus microscope attached to an Olympus DP70 digital camera (Japan) from four regions 1) the hippocampus, including CA1, CA3 and DG; 2) the CeA and LA amygdaloid areas; 3) the PV, CM, DL and MD thalamic nuclei, and 4) the MHb and LHb. The analysis of the hippocampus was further divided by layers and measurements were taken in the oriens, pyramidal and radiatum layers of CA1; the oriens, pyramidal, lucidum and radiatum layers of CA3 and the granular and molecular layers of the medial blade (mbDG) and lateral blade (lbDG) of the DG and the hilus. The image analysis software Image-J 1.43 (http://rsb.info.nih.gov/ij/) was used to assess greyscale intensity levels using circular regions of interest (ROIs). An average of Arc intensity levels from three histological sections between bregma − 2.50 and − 3.36 for all regions. For the CM and PV, ipsilateral and contralateral sides were not taken into account.

### Statistics

The statistical computer package program PASW Statistics 17.0 (SPSS) was used to process the data. Analyses of Arc intensity levels/mm^2^ were conducted with a mixed ANOVA independent for each brain region. This corresponds to one between-group factor, the *TREATMENT* (sham or ICSS) and one within-group factor, the *HEMISPHERE* (ipsilateral or contralateral to electrode placement). A second within-group factor was considered for the hippocampal regions, the *LAYER* (3 for CA1, 4 for CA3, 5 for DG; see 2.3.3). Student’s *t* test was applied to analyze the ICSS effects on Arc expression in the PV and CM nuclei. Additionally, in order to study the underlying structure of the regions where significant ICSS effects on Arc expression were observed principal components analysis (PCA) with *oblimin* method of rotation was conducted. The α level for all tests was set at.05.

## Results

### ICSS treatment

The mean values (± SD) of the ICSS variables of the ICSS group were: OI (77.14 ± 42.31 µA), highest response rate (73.92 ± 13.30 responses/min), treatment duration (50.42 ± 8.97 min) and number of responses in the treatment session (3052.42 ± 233.60 lever pressings). Correlation analyses showed no relationship between the ICSS variables and Arc levels in any of the brain areas evaluated.

### Arc protein expression in different memory system areas following ICSS

We analyzed the influence of unilateral MFB-ICSS on different memory-related brain areas using Arc protein as a marker of early synaptic plasticity and examined both the ipsilateral and contralateral hemispheres. We compared the expression of Arc protein in both the MFB-ICSS and Sham groups in discrete regions of the hippocampus, amygdala, thalamus and the habenula 4.5 h after treatment.

#### Hippocampus

**Fig. 1:**
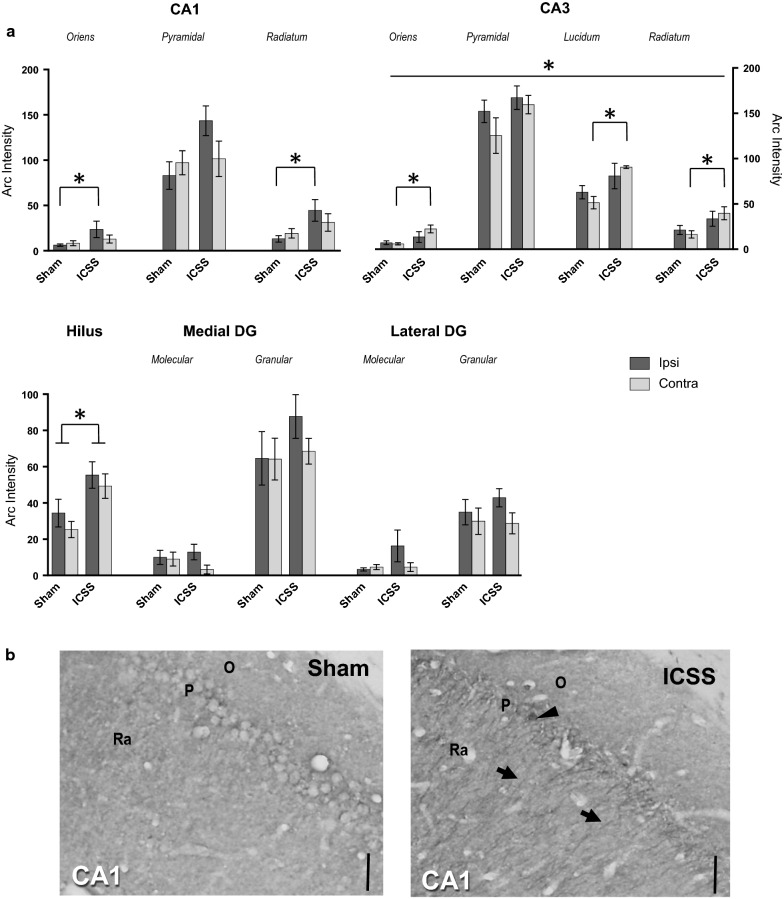
Arc protein expression in rat hippocampal subfields after MFB-ICSS treatment. **a** Mean immunohistochemical intensities in each analyzed layer from CA1, CA3 and DG hippocampal subfields in the ipsilateral and contralateral hemispheres.*p  <.05 versus Sham group. Standard errors are indicated with error bars. **b** Representative immunohistochemestry image of Arc protein expression in the CA1 subfield from one subject from the Sham and MFB-ICSS groups. (x400, scale bar 25 µm; stereotaxic coordinates AP – 3.24 bregma). Black arrows and arrowhead indicate Arc immunoreactive cytoplasmatic prolongations and cell body, respectively (Ra, radiatum; La, lacunosum; P, pyramidal; O, oriens)

ANOVA analysis revealed a statistical significant increase of Arc protein expression in the ICSS group compared to the Sham group in the CA1 region dependent on both hemisphere and layer [*TREATMENT *× *LAYER *× *HEMISPHERE*: F_(2,30)_ = 3.68; *p* = .037] (see Fig. 1a, b). Further simple effects analysis showed significant effects in the ipsilateral hemisphere (*p* = .05), however, this was dependent on layer [*TREATMENT *× *LAYER*: F_(3,48)_ = 2.68; *p* = .007]. Thus ICSS increased Arc expression in the ipsilateral stratum oriens [F_(1,15)_ = 4.93; *p *= .042] and radiatum [F_(1,15)_ = 5.75; *p *= .03] layers but not in the pyramidal layer (*p *= .07). In contrast, no effects of the ICSS were observed in the contralateral side (*p *= .755) in any layer (oriens, *p *= .360; radiatum, *p *= .319; pyramidal, *p *= .998).

The increase in Arc protein expression in the CA3 region of the MFB-ICSS group compared to Sham [F_(1,15)_ = 5,65; *p *= .031] was independent of both hemisphere and layer [× *HEMISPHERE*: *p *= .442; × *LAYER*: *p *= .669] (Fig. 1a). However, since certain hemisphere differences were observed depending on the layer [*HEMISPHERE *× *LAYER*: F_(3,48)_ = 3.9; *p *= .034], simple effects for each hemisphere were studied. Analysis showed significant ICSS effects in the contralateral side (*p *= .038), but not in the ipsilateral (*p* = .095). Specifically, an Arc increase was observed in the contralateral stratum oriens (*p *= 0.004), lacunosum (*p *= .05) and radiatum (*p *= .016), but not in the pyramidal layer (*p* = 0.289).

Finally, MFB-ICSS treatment had no significant overall effect on the dentate gyrus [F_(1,15)_ = .663; *p *= .428] and no significant interactions (× *HEMISPHERE*: *p *= .275; × *LAYER*: *p *= .250]. However, a significant difference in Arc protein increase between ICSS and Sham groups was observed in the Hilus [F_(1,15)_ = 4.73; *p *= .046] independent of the hemisphere (*p *= .683) and a tendency to significance in the ipsilateral molecular layer of lbDG (*p *= .094), but not in the contralateral (*p *= .885) was found (Fig. 1a).

#### Amygdala

**Fig. 2:**
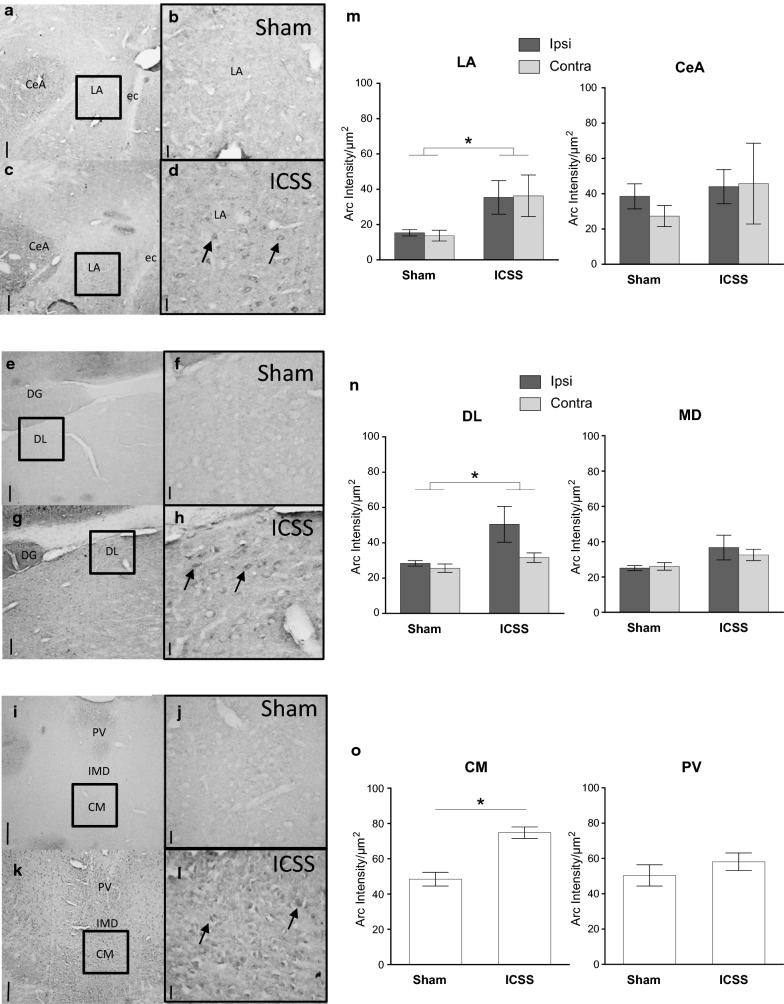
Arc protein expression in amygdala nuclei (**m**) and thalamic nuclei (**n** and **o**) after MFB-ICSS treatment. Representative immunohistochemistry images of Arc protein expression in the lateral amydala (LA), dorsolateral (DL) and central medial (CM) thalamic nuclei from the Sham (**a** and **b**, **e** and **f**, **i** and **j**) and MFB-ICSS (**c** and **d**, **g** and **h**, **k** and **l**) groups. (scale bar 100 µm in **a**, **c**, **e**, **g**, **i** and **k**, and 25 µm in **b**, **d**, **f**, **h**, **j**, and **l**; stereotaxic coordinates between − 2,50 and – 3.36 AP to Bregma). Arrows indicate Arc immunoreactive cell bodies. (CeA, central amygdala; ec, external capsule; DG, dentate girus; PV, paraventricular thalamic nuclei; IMD, intermediodorsal thalamic nucleus). Bar charts show the mean Arc expression levels in the LA and CeA (**m**) and in the DL and mediodorsal (MD) thalamic nuclei (**n**) from ipsi and contra lateral hemispheres of sham and MFB-ICSS treated groups. **o** mean Arc expression levels in CM and PV thalamic nuclei of sham and MFB-ICSS groups. Standard errors are indicated with error bars

Since the Box test for equality of covariance matrices was significant (*p* = .010) in the LA, a conservative F correction was therefore applied. ANOVA revealed that the *TREATMENT* had a significant effect [F_(1,7)_ = 6,51; *p *= .038], independent of hemisphere (*p *= .623). No significance was found in the CeA (*TREATMENT*: *p *= .471; *TREATMENT *× *HEMISPHERE*: *p* = .407) (see Fig. 2m).

#### Thalamus

Student’s t-test showed a significant increase in Arc protein levels after MFB-ICSS treatment in the CM [t _(9)_ = 4.59; *p* = .001] but not in the PV [t _(13)_ = 0.910; *p* = .379] thalamic nuclei (see Fig. 2O). In the DL nucleus, the *TREATMENT* was found to have a significant effect [Box test *p* = .027; F _(1,5)_ = 7.34; *p *= .042] independent of hemisphere (*p *= .076). In the MD, no significant effect of the *TREATMENT* factor [Box test *p *= .04; F _(1,5)_ = 4.013; *p *= .101], nor the *HEMISPHERE* (*p *= .467) or their interaction (*p* = .279) were detected (see Fig. 2n).

#### Habenula

**Fig. 3:**
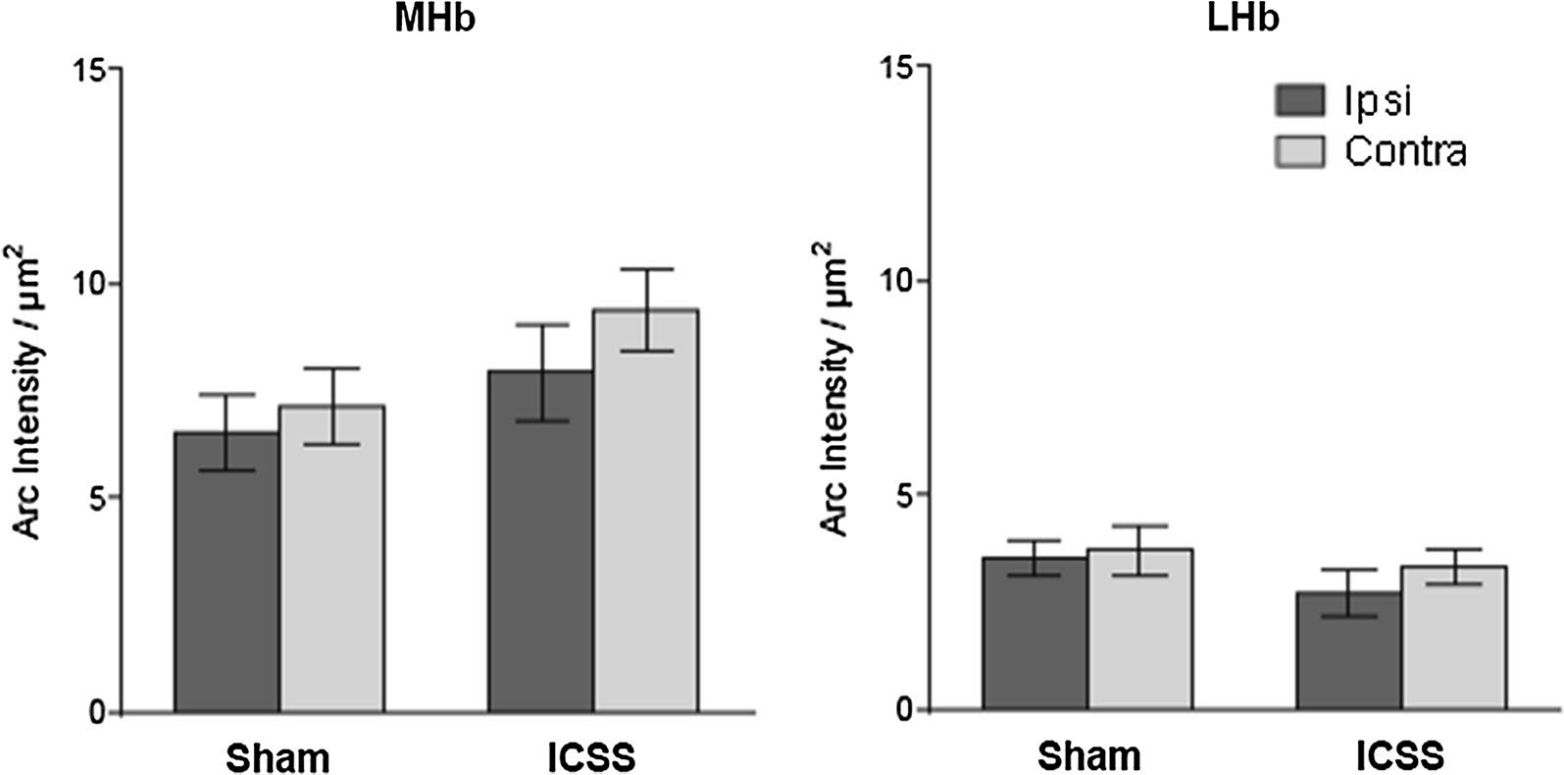
Arc protein expression in the habenula complex after MFB-ICSS treatment. Bar charts show the Arc expression levels of sham and MFB-ICSS treated groups in the medial (MHb) and lateral (LHb) habenula from ipsi and contra lateral hemispheres

ANOVA analysis revealed that the *TREATMENT* had no significant effects on either the medial [F_(1,17)_ = .331; ***p***= .573] or lateral [F_(1,17)_ = 1.815; ***p***= .196] habenula, or its interaction with the *HEMISPHERE* (MHb, ***p***= .847; LHb, ***p***= .189) (Fig. 3).

#### Relations among Arc expression in different brain regions

Principal component analysis (PCA) was carried out on regions where significant effects of MFB-ICSS were observed, in order to identify whether or not there were any subsets of functionally-related regions to the expression of Arc. First of all, in order to reduce the variables of the analysis, we identified regions with significant correlation values between the ipsilateral and contralateral side (in relation to the electrode implantation) and in which the variable hemisphere did not influence the effects of the treatment. These areas were included in the PCA as the Arc intensity average of both hemispheres (LA, Hilus and DL variables) or in the region (CM). The CA1 and CA3 neurite layers also showed significant MFB-ICSS effects but with very different behavior in each of the hemispheres, however, this was not the case for the pyramidal layers. In this case, the CA1 and CA3 neurite layers were included in the PCA as Arc intensity average in oriens and radiatum layers from ipsilateral CA1 (CA1-neurite-ipsi) and in oriens, radiatum and lacunosum layers from contralateral CA3 (CA3-neurite-contra) variables. Therefore, the PCA included the following variables: LA, H, DL, CM, CA1-neurite-ipsi and CA3-neurite-contra.

**Fig. 4:**
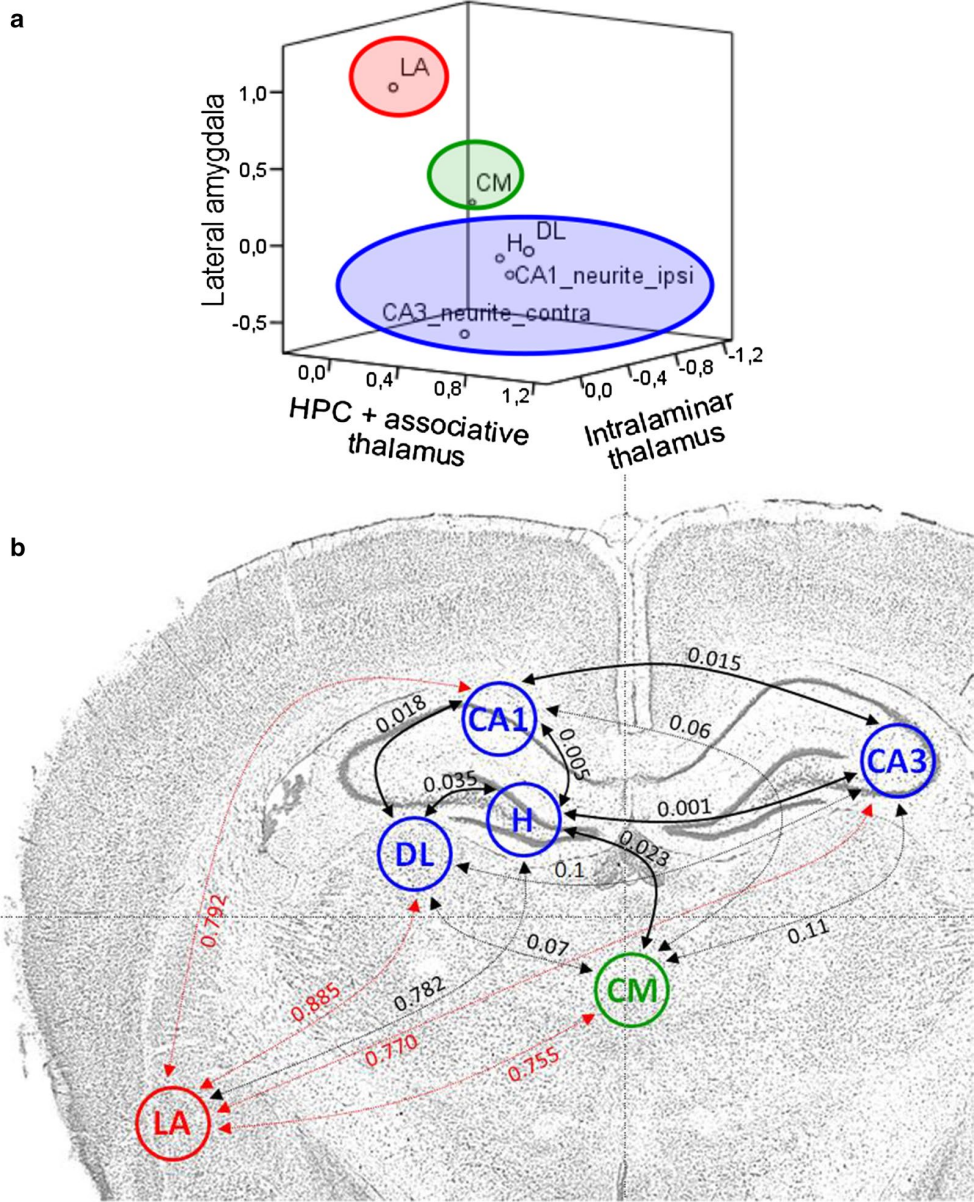
Component graphic in rotated space showing brain regions with Arc-related plasticity as a consequence of the MFB-ICSS treatment.** a** Three-dimensional representation of the regions where the MFB-ICSS has significant effects, according to the correlation between the observed Arc protein levels. Three components were extracted, the first composed of different fields of the hippocampus and the dorsolateral associative nucleus of the thalamus (blue); the second by the lateral amygdala (red) and the third by the nonspecific thalamic nucleus CM (green) (Bartlett’s sphericity test, *p* = .005).** b** Anatomical representation showing *p* values of correlations between the different areas according to the observed Arc protein levels 4.5 h post-MFB-ICSS

Bartlett’s sphericity test was statistically significant (*p* = .005). A 3-component model explained 91.83 percent of the variance. Figure 4 shows rotated factor loading. Factor 1 included four items (H, neurite layers of contralateral CA3 and ipsilateral CA1 and the DL thalamus), and could be labeled “hippocampus + associative thalamus”. Factor 2 consisted of the lateral amygdala and the unspecific CM thalamus mainly loaded onto the third factor. Factor correlations were low for factor 2 (r ≤ .29) and moderated between factor 1 and 3 (r = .59).

## Discussion

This study analyzes Arc protein expression in specific memory-related brain regions including the hippocampus, amygdala, thalamus and habenula through which ICSS of the MFB could exert its enhancing effect on memory. Results showed that unilateral MFB-ICSS, delivered with the same parameters with which it facilitates memory, upregulated Arc protein expression in the CA1, CA3 and hilus hippocampal regions, lateral amygdala, CM and DL thalamic nuclei. A differential hemispheric synaptic plasticity response was observed depending on the analyzed region.

Present results support our previous findings in that they show an overexpression of Arc protein in the neurite layers of the ipsilateral CA1 hippocampal region 4.5 h after MFB-ICSS [22], which is an intermediate time point for Arc protein expression changes [38]. This expression did not increase after the same treatment in contralateral CA1. To support our results further, structural plasticity changes, such as increased spine density and increased size and branching complexity in CA1 dendritic arborizations were also detected, specifically in the ipsilateral CA1 region of the hippocampus, 3 days and 20 days post-MFB-ICSS [6]. In contrast, Arc expression in the CA3 subfield showed predominance in the contralateral hemisphere which indicates that the lack of MFB-ICSS effects previously reported on ipsilateral CA3 is a hemisphere-dependent effect. Previous results of c-Fos expression showing the bilateral activation of CA1 and CA3 after unilateral MFB-ICSS [10] suggest that the hemispheric differences are not a result of higher contralateral hemispheric activation but rather a higher plasticity response. Therefore, differential expression patterns between these two proteins may be a sign of the greater specificity of Arc protein expression in terms of plasticity. Interestingly, hemispheric asymmetry was found in CA3-CA1 pyramidal neuron synapses [39]. This asymmetry has also been observed in long-term memory, where only left CA3 silencing impaired performance in an associative spatial long-term memory task, whereas right CA3 silencing had no effect, suggesting that memory could be routed via distinct left–right pathways within the mouse hippocampus [40]. In the DG, although a global effect was not observed, a significant bilateral increase in Arc expression in the hilus was found, as well as a tendency towards significance in the ipsilateral molecular layer of the lbDG. This reflects sub-regional treatment-induced differences related to synaptic plasticity, similar to previous studies [22].

It has been shown that an analogous MFB-ICSS treatment induces expression changes of different synaptic plasticity-related genes such as Arc and BDNF in the rat amygdaloid complex [19]. However, Arc expression was not analyzed at the protein level in specific amygdala nuclei after MFB-ICSS. Boundaries of expression corresponding to cytoarchitectonically defined amygdala subnuclei have been described in basal conditions [41] and here we observed a significant increase in Arc protein expression 4.5 h after MFB-ICSS treatment in the LA nucleus, whereas no effects were observed in the CeA nucleus. Interestingly, Partin et al. [42] described notable differences in gene expression patterns in the CeA and the LA nuclei. This differential response after MFB-ICSS in LA supports additional experiments in our laboratory in which a differential acetylcholinesterase activity linked to synaptic plasticity events in the LA but not in CeA regions was observed in MFB-ICSS treated lesioned rats. At the behavioral level, MFB-ICSS completely reversed the impairment in the acquisition of an active avoidance task caused by amygdala lesions [15]. There are also functional differences between these two amygdalar regions. The LA is an essential region for fear memory storage whereas the CeA does not seem to be required for the manifestation of instrumental active avoidance conditioned responses [43]. In agreement with our results, the immediate-early gene Arc has been recognized as a molecular marker for the LA neuronal ensemble recruited during fear learning in mice [44]. Regarding the observed MFB-ICSS hemisphere effects, it is important to point out that while an ipsilateral and contralateral pattern of Arc expression was observed in CA1 and CA3 respectively, a bilateral increased Arc expression was obtained in the LA, in a way that is consistent with the previous cited works, since no hemispheric effects were reported.

Regarding the thalamus, the effects of MFB-ICSS on thalamic synaptic plasticity events related to learning and memory have not been described and few studies have explored this area after electrical stimulation. For instance, LTP and/or LTD have been observed in the anterior thalamus after stimulation of direct and indirect hippocampal projections [45] and Arc protein synthesis has only been explored in relation to the thalamus role as a sensory relay [46, 47]. Present results show that MFB-ICSS induces bilateral Arc expression changes in specific thalamic nuclei including the DL and CM nuclei, whose role in memory may account for improvements in learning tasks. The DL nucleus acts in concert with the anterior nuclei and may serve an important integrative purpose for spatial learning systems [48–50]. The CM nucleus, strongly connected to the mPFC, is likely to be involved in working memory and/or memory consolidation through its modulation of vigilance states [25, 51]. Contrary to expectations, under present conditions MFB-ICSS did not increase Arc protein expression in the DM and PV nuclei, even though they also seem to participate in memory and executive function. The MD nucleus may contribute more to adaptive decision-making, as it is connected to the orbital prefrontal cortex and basolateral amygdala [52]. The PV nucleus, being part of the dorsal midline group, seems to contribute to viscero-limbic functions, reward and defensive behavior, and is a relay station between specific parts of the prefrontal and cingulate cortex, the striatum, and the CeA [25, 53, 54]. Overall, these results indicate that the thalamic nuclei most directly related to memory may mediate the facilitation effects of the MFB- ICSS on memory to some extent. Furthermore, in the same way that MFB-ICSS has proven to potentiate both explicit and implicit memory, the thalamus has also been linked to both memory systems involved. Thus, recent enhancement of c-Fos activity and the alpha4-nicotinic acetylcholine receptor in the hippocampus was observed after central thalamic DBS treatment [55], and expression of genes related to protein synthesis, maturation and degradation are increased in thalamic neurons that project to the LA after fear conditioning [56].

Our results also suggest the existence of certain functional subsystems in regions showing Arc-related plasticity as a consequence of the MFB-ICSS treatment. On the one hand, it is worth noting the autocorrelation between the different parts of the hippocampus and also their functional connection with the DL [57, 58], but not with the CM nucleus of the thalamus. The notion that the latter involves a second subsystem or component is supported by the described lack of direct anatomical connections between the intralaminar thalamus and the hippocampus [25]. The associative LA is a substrate of action of the MFB-ICSS functionally independent of the hippocampus-DL and CM subsystems. However, since Arc and other plasticity and neuroprotection genes are upregulated in the amygdala before the 4,5 h time point (90 min post MFB-ICSS) [19], we cannot rule out that the activation of the amygdala and hippocampus, or thalamus, could be sequential, yet still related (Additional file 1).

The contribution of the habenular complex to ICSS has been examined in different studies but contradictory results have been observed. While Morissette et al. [59]showed that electrolytic lesions of the habenula attenuate brain stimulation reward, Gifuni et al. [60] showed that neurotoxic lesions of the LHb neurons do not alter the reward-enhancing effect of D-amphetamine in ICSS. Duchesne et al. [61] concluded that mesohabenular dopamine is not an important contributor to brain stimulation reward. Moreover, though recent evidence indicates that the habenular complex plays a role in learning and memory [62], no studies have looked at the effects of ICSS on plasticity-related protein expression in the habenula. Our findings show that MFB-ICSS did not enhance habenular Arc protein expression levels (or c-Fos protein expression- data not shown), at least at the time point analyzed. This supports the idea that the habenular complex may not be involved in the anatomical circuit activated by ICSS.

Finally, all these results taken together support the hypothesis that the stimulation of the reward system activates neural plasticity mechanisms in memory related areas and point MFB as a promising target for memory enhancing treatments. However, an aspect to consider is whether the results obtained with MFB-ICSS could be expected when MFB-stimulation was not self-administered, as it would be in a clinical setting. Although there are no antecedents comparing Arc protein levels caused by passive versus active administration, studies have reported that this variable slightly affects the induction c-Fos expression in the ipsilateral LH, but does not affect other brain memory-related regions [63]. Moreover, Chergui et al. [64] considered that, more than being self-administered or not, a critical parameter of the stimulation could be the temporal organization of the action potentials they generate. Similarly, although at the behavioral level both procedures—self-administered and experimenter-administered- have been shown to facilitate memory [65], the reinforcement component would correlate with the efficiency to potentiate memory [66]. Further studies should be performed to elucidate the differences between these two stimulation paradigms more thoroughly.

## Conclusions

Overall, MFB-ICSS upregulates the expression of Arc protein in specific memory related areas including the CA1, CA3 and hilus hippocampal regions, the lateral amygdala and the dorsolateral and central medial thalamic nuclei which showed differential hemispheric response to the treatment. Our findings back up the idea that multiple, separate brain systems, could operate in parallel to support MFB-ICSS behavioral effects with distinct purposes during memory consolidation. Further studies may be performed not only to rule out the contribution from any other long-term storage related areas, such as the medial prefrontal cortex, but also to analyze whether ICSS may reverse the impact that certain brain lesions may have on cognition and memory, by potentiating other functionally related areas.

## Additional file

**Additional file 1.** In the Additional file 1, titled DATA BASE_Arc, it is included all raw data of Arc intensity levels in all subregions from amygdala, thalamus, hippocampus and habenula regions in ipsilateral (i) and contralateral (c) hemispheres in the sham group (0) and ICSS group (1) according to the described methodology (each data is the average of Arc intensity levels from three histological sections from each rat).

## Abbreviations

Arc: activity-regulated cytoskeleton-associated
CM: central medial
Ce: central nucleus
DBS: deep brain stimulation
DL: dorsolateral
ICSS: Intracranial Self-Stimulation
F-DBS: forniceal DBS
LA: lateral amygdala
lbDG: lateral blade of DG
LHb: lateral habenula
LH: lateral hypothalamus
mbDG: medial blade of the DG
MFB: medial forebrain bundle
MHb: medial habenula
MD: mediodorsal
OI: optimal intensity
PV: paraventricular
PBS: phosphate buffer saline
PCA: principal components analysis
ROIs: regions of interest
RSC: retrosplenial cortex
